# Increasing Fertilization Efficiency of Biomass Ash by the Synergistically Acting Digestate and Extract from Water Plants Sequestering CO_2_ in Sorghum Crops

**DOI:** 10.3390/molecules29184397

**Published:** 2024-09-16

**Authors:** Zdzisława Romanowska-Duda, Regina Janas, Mieczysław Grzesik

**Affiliations:** 1 Faculty of Biology and Environmental Protection, University of Lodz, Banacha 12/16, 92-237 Lodz, Poland; 2 Department of Cultivar Testing, Nursery and Gene Bank Resources, The Institute of Horticulture—National Research Institute, Konstytucji 3 Maja 1/3, 96-100 Skierniewice, Poland; regina.janas@inhort.pl (R.J.); mieczyslaw.grzesik@inhort.pl (M.G.)

**Keywords:** sorghum, Jerusalem artichoke, ash, plant growth, physiological activity, fertilization, synergism, *Spirodela polyrhiza*

## Abstract

The utilization of biomass ash in sustainable agriculture and increasing its fertilizing efficiency by biological agents, potentially sequestering CO_2_, have become important issues for the global economy. The aim of this paper was to investigate the effects of ash from sorghum (*Sorghum bicolor* L. Moench) and Jerusalem artichoke (*Helianthus tuberosus* L.) biomass, a biogas plant digestate, and a *Spirodela polyrhiza* extract, acting alone or synergistically, on soil fertility and the development, health and physiological properties of sorghum plants. The results show novel information concerning differences in the composition and impact of ash, depending on its origin, soil properties and sorghum plant development. Sorghum ash was more effective than that from Jerusalem artichoke. Ash used alone and preferably acting synergistically with the digestate and *Spirodela polyrhiza* extract greatly increased soil fertility and the growth, biomass yield and health of sorghum plants. These improvements were associated with an increased chlorophyll content in leaves, better gas exchange (photosynthesis, transpiration, stomatal conductance), greater enzyme activity (acid and alkaline phosphatase, RNase, and total dehydrogenase), and a higher biomass energy value. The developed treatments improved environmental conditions by replacing synthetic fertilizers, increasing the sequestration of CO_2_, solving the ash storage problem, reducing the need for pesticides, and enabling a closed circulation of nutrients between plant and soil, maintaining high soil fertility.

## 1. Introduction

The management of biomass ash and its use in agricultural production together with other ecological fertilizers that help to improve its efficiency have become one of the most important issues in a sustainable global economy. Therefore, the need to develop methods to improve the fertilizing effectiveness of such ash and to introduce technologies for its use in order to improve plant productivity in the context of environmental protection and CO_2_ sequestration has become particularly important [1,2]. The need to reduce greenhouse gas emissions necessitates a gradual replacement of fossil fuels with renewable energy sources, including biomass burning, which leads to greatly increasing amounts of ash. As the combustion of biomass becomes a prospective branch of energy production and an alternative to fossil fuels, the increasing volume of ash poses a serious problem for its economic and environmentally friendly management [3]. Biomass is the preferred source of energy as it can be used in various ways to generate energy. According to the World Bioenergy Association [4], the energy obtained from biomass constitutes 59.2 EJ/yr or 10.3% of global energy production. It is estimated that about 480 million Mg of ash from biomass combustion or co-firing can be generated every year around the world, assuming that the amount of biomass burnt is 7 billion Mg/year [5,6]. Given the environmental and agricultural benefits, its use as an alternative to artificial fertilizers in plant production seems to be the most rational choice. The chemical composition of biomass ash, dominated by macro- and microelements and the lack or low concentrations of toxic elements, depending on the burnt plant species, makes it suitable for agricultural use. The possibility of returning the elements contained in the ash from the burnt biomass to the soil as a fertilizer indicates the legitimacy of its utilization in circular agricultural production [7]. In this way, a significant part of the elements taken up by the plants returns to the soil in the ash fertilizer, closing their circulation and preventing soil depletion [7,8]. Numerous reports show beneficial amounts of elements in the ash from biomass of selected plant species [3,5,6,8]. Some of them indicate that its use in proper doses may have a deacidifying effect and can improve the physicochemical properties of light soils [9,10,11]. However, ash from biomass can show significant variations in properties and structure, depending on the kind and origin of the burnt biomass, which may influence the fertilizing efficiency [10]. Biomass ash can also contain toxic substances, including heavy metals, absorbed by long-growing trees, which makes their use in agriculture impossible [8]. Thus, the potential use of a given ash as a fertilizer should be considered individually depending on the biomass origin and the technology of burning it as well as on the plants to be fertilized. The majority of relevant literature concerns the effects of ash on the composition of macroelements in the soil and the use of ash from burnt wood in the cultivation of forest trees, excluding optimization of dose efficiency with other compounds [12]. Santalla et al. [3] and Vassiliev at al. [5] indicated the possibility of using wood ash for the fertilization of forest plants and some other crops. Jagodzinski et al. [13] demonstrated a positive effect of wood ash on the growth of the aquatic plant lesser duckweed (*Lemna minor* L.). Buss et al. [14] stated that the application of wood ash to forest and agricultural soils can provide nutrients and increase soil pH. However, it can also change the soil chemistry rapidly and temporarily, sometimes resulting in reduced growth of plants and leaching of potassium [14]. The data concerning the use of ash from annual plants, possibly free of toxic materials, and its impact on plant health and productivity are still very limited. They are included, for example, in the works by Piekarczyk et al. [15], Ciesielczuk et al. [16] and Meller and Bilenda [10] and show the beneficial influence of ash from some burnt crops on soil enrichment with several nutrients.

Although the literature data cited above indicate the possibility of using various kinds of ash as fertilizer, little attention has been paid to increasing their fertilizing efficiency through the use of additional ecological biological agents in a synergistic system, taking also into account the possibility of CO_2_ sequestration. Greenhouse gas emissions can be effectively reduced by improving the fertility of depleted soils with ash, increasing the yield of the biomass to be used as an alternative to fossil fuels, and sequestering CO_2_ by the growing plants and the fertilizers used. The implementation of these innovative techniques is crucial in dealing with the challenges imposed by recent environmental changes [17]. The literature data so far has focused on the selection of biofertilizers best able to increase plant growth, which is a decisive criterion for recommending them in crop cultivation. However, little attention has been paid to the additional possibility of reducing greenhouse gas emissions that may be associated with sorghum crops. Therefore, this new approach to intensifying the growth of sorghum (*Sorghum bicolor* L. Moench) is undertaken in the presented research, taking into account the synergistic effects of ash, a digestate from a biogas plant and an extract from the aquatic plant common duckweed (*Spirodela polyrhiza* L. Schleid), which in combination should increase plant growth and additionally sequester CO_2_ from the atmosphere.

Sorghum, the fifth most cultivated cereal in the world, is increasingly seen as a prospective plant for multidirectional use and an alternative to corn in some regions, as it can be cultivated for silage and energy purposes on poor soils, under the conditions of scanty precipitation and changing climate. It is also considered a promising energy crop because it grows in 5–6 months and is capable of abiotic stress tolerance. This species shows great capacity for CO_2_ sequestration (50 g C m^−2^ day^−1^), has higher concentrations of sugars in its stalks, lower fertilizer and pesticide requirements, and is cultivable under diverse climates. It is also assumed that sorghum, as a bioenergy crop, could sequester soil carbon at the rate of 0.6 t C ha^−1^ year^−1^, apart from the CO_2_ absorbed by the plants from the atmosphere in the process of photosynthesis. The global production of bioethanol from sorghum ranges from 3 to 9 m^3^ ha^−1^, and 762.6 cm^3^ of biogas can be manufactured from 1 g of its dry biomass [18,19,20].

Biomass ash, besides having possibly high fertilizing properties, also has a CO_2_ sequestration potential [7]. It is estimated that 14.5 g of CO_2_ can be fixed by 1 kg of biomass fly ash and 16.5 g CO_2_ by 1 kg of biomass bottom ash used during plant cultivation, and also 19.7 g of CO_2_ by 1 kg of biomass bottom ash not used in plant cultivation [1]. In those cases, CO_2_ combines with calcium or magnesium minerals contained in the ash to form stable carbonate minerals such as calcium carbonate (CaCO_3_) or magnesium carbonate (MgCO_3_). This reaction depends on the temperature, moisture content of the soil and the calcium content in ash, which is determined by its origin, as the presented results show [21].

Digestate from biogas plants, highly recommended as bio-fertilizer by Romanowska-Duda et al. [22] and Chojnacka and Moustakas [23], is obtained from plants assimilating atmospheric CO_2_ and has also a promising potential for the direct capture of CO_2_ from the air [19,24]. The use of digestate to create organic fertilizers is a promising choice and fills the gap in the formulation of full-value fertilizers [23]. The additional capacity for sequestering CO_2_ from the air would be an additional advantage of its use in sustainable agriculture [19,24]. 

The aquatic plant *Spirodela polyrhiza* can be used to transfer N and P from eutrophic water bodies to field or sorghum crops, making it a very cost-effective bio-fertilizer [25]. It possesses the highest relative growth rates among plants, prevents the release of CO_2_ from the soil into the atmosphere, and produces multiple products and services of human interest. It is significantly efficient in plant fertilization due to a high nutrient content promoting the growth and health of plants. Even when cultivated in small quantities, it is highly efficient at removing CO_2_ from the atmosphere [26,27,28]. The validity of undertaking research on the use of a *Spirodela polyrhiza* extract as a bio-fertilizer is emphasized by the research by Buono et al. [29]. They showed a beneficial effect of the extract from *Lemna minor*, an aquatic plant belonging to the same *Lemnaceae* family as *Spirodela*, on the kernel germination, biomass, leaf area, pigment content and vigor of maize plants. To the best of our knowledge, there is no information regarding the mentioned technology nor any comparisons of similarities or differences in the composition and fertilizing efficiency of ash from sorghum (*Sorghum bicolor* L. Moench) and Jerusalem artichoke (*Helianthus tuberosus* L.), and the possibility of increasing the beneficial effects of such ash on soil fertility and sorghum growth by supplementing it with digestate and *Spirodela polyrhiza* extract remains unknown. Therefore, the research presented below attempted to optimize the fertilizing efficiency of these kinds of ash from the perspective of fertilizing sorghum energy crops with biological agents assimilating atmospheric CO_2_ with a view to reducing environmental pollution.

The aim of the presented research was to assess the composition of ash from burnt sorghum and Jerusalem artichoke biomass, a digestate from a biogas plant, and a *Spirodela polyrhiza* extract, and to investigate their synergistic impact on soil quality improvement and the physicochemical properties, growth, health and yield of plants in order to optimize the fertilizing efficiency of such dust in sorghum cultivation. 

## 2. Results

### 2.1. Elemental Composition of Soil and the Applied Bio-Fertilizers 

The ash used for soil fertilization contained significant amounts of macro- and micronutrients which in most cases were greater than those present in the soil. The quantitative compositions of elements in the sorghum (*Sorghum bicolor* L. Moench) ash and the Jerusalem artichoke (*Helianthus tuberosus* L.) ash were different, and the weight per unit volume of the former was half that of the latter. The percentage of ash in the biomass ranged from 3.88% to 3.78%. The sorghum ash contained more N (10%), P (4%), K (5%), Mg (14%), Cu (6%) and Zn (20%), and less Ca (30%) than the ash from Jerusalem artichoke. The concentrations of unburnt C and of Fe, Mn and B, and also the pH were comparable in the two kinds of ash (Table 1).

The fertilization with ash and digestate resulted in a significant increase in the concentration of elements in the podzolic soil to the extent depending on the ash origin. When the ash from sorghum was used in this mixture, the total content in soil of N, P, K and Mg increased and was higher by 20, 15, 4 and 14%, respectively, while the Ca content was 4% lower than after the treatment with the Jerusalem artichoke dust. The total amounts of Fe and Mn in the soil were increased to a similar extent, while the amounts of Cu, Zn and B did not change significantly after ash application (Table 1). 

The amounts of macro- and microelements in the biomass of *Spirodela polyrhiza* were lower than in the ash and much higher than in the soil, which proves the high fertilizing value of these aquatic plants. These plants contained an extremely high boron content in their biomass, which was five to six times higher than in the ash and seven times higher than in the soil. Better fertilizing properties were also exhibited by the biogas plant digestate, which contained significantly high amounts of macro- and microelements, thus positively affecting plant development. However, their concentrations may change in subsequent cycles of biomass digestion into methane. Detailed information on the elemental composition of ash and the soil before and after fertilization with the bio-fertilizers, and also the composition of the waste from the biogas plant and the *Spirodela polyrhiza* extract, are given in Table 1.

In relation to the above-mentioned elements and their quantities, the amounts of available nutrients in the soil greatly increased after the treatment with ash and digestate. The concentration of available K and Mg was higher by 3 and 8%, respectively, after fertilization with sorghum ash than with Jerusalem artichoke dust. The Ca content was 5% lower after the application of sorghum ash compared to the treatment with Jerusalem artichoke ash. The amounts of available organic C and N (NO_3_^2−^) and P in the soil increased to a similar extent regardless of the type of applied ash, as did the pH and salinity (Table 2). 

### 2.2. Effect of the Applied Bio-Fertilizers on Sorghum Growth and Biomass Yield 

**Figure 1:**
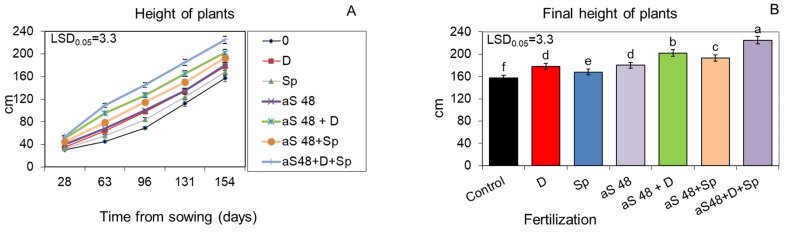
Growth kinetics (**A**) and final height (**B**) of sorghum plants fertilized with sorghum ash, in doses of 48 q ha^−1^ (aS 48), applied to the soil alone or together with the biogas plant digestate (D; 30 m^3^ ha^−1^) and/or with the *Spirodela polyrhiza* extract (Sp; 7 L ha^−1^) as foliar spray. The LSD was calculated at the significance level of *p* = 0.05. Means with the same letters are not significantly different, according to Newman–Keuls multiple range test at an alpha level of 0.05. Error bars show mean ± SD of three independent replicates.

**Figure 2:**
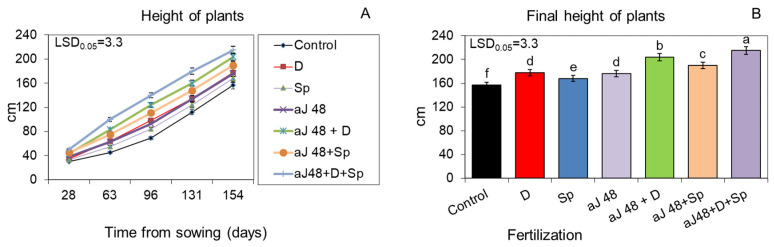
Growth kinetics (**A**) and final height (**B**) of sorghum plants fertilized with Jerusalem artichoke ash, in doses of 48 q ha^−1^ (aJ 48), applied to the soil alone or together with the biogas plant digestate (D, 30 m^3^ ha^−1^) and/or with the *Spirodela polyrhiza* extract (Sp; 7 L ha^−1^) as foliar spray. The LSD was calculated at the significance level of *p* = 0.05. Means with the same letters are not significantly different, according to Newman–Keuls multiple range test at an α level of 0.05. Error bars show mean ± SD of three independent replicates.

**Figure 3:**
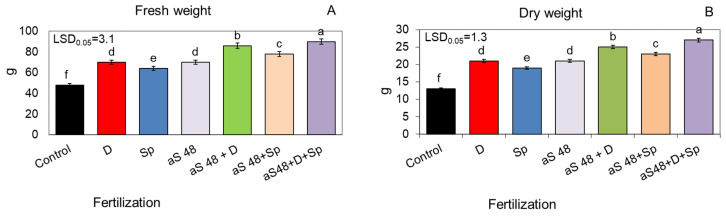
Fresh (**A**) and dry (**B**) weight (per one plant) of sorghum plants fertilized with sorghum ash, in doses of 48 q ha^−1^ (aS 48), applied to the soil alone or together with the biogas plant digestate (D; 30 m^3^ ha^−1^) and/or with the *Spirodela polyrhiza* extract (Sp; 7 L ha^−1^) as foliar spray. The LSD was calculated at the significance level of *p* = 0.05. Means with the same letters are not significantly different, according to Newman–Keuls multiple range test at an α level of 0.05. Error bars show mean ± SD of three independent replicates.

**Figure 4:**
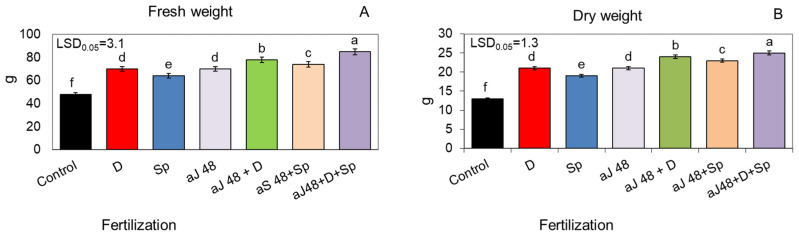
Fresh (**A**) and dry (**B**) weight (per one plant) of sorghum plants fertilized with Jerusalem artichoke ash, in doses of 48 q ha^−1^ (aJ 48), applied alone or together with the biogas plant digestate (D; 30 m^3^ ha^−1^) and/or with the *Spirodela polyrhiza* extract (Sp; 7 L ha^−1^) as foliar spray. The LSD was calculated at the significance level of *p* = 0.05. Means with the same letters are not significantly different, according to Newman–Keuls multiple range test at an α level of 0.05. Error bars show mean ± SD of three independent replicates.

The ash obtained from burnt sorghum and Jerusalem artichoke biomass, applied to the soil at the dose of 48 q ha^−1^, significantly increased plant growth kinetics, which was evidenced by a markedly higher shoot height during the entire vegetative season than in the unfertilized control (Figure 1A,B and Figure 2A,B). However, in all the variants, the sorghum ash had a slightly more beneficial effect on plant growth than the Jerusalem artichoke ash. This relationship was maintained even when the positive effect of the tested ash from both plant species was additionally reinforced by the additional enrichment of the soil with the biogas plant waste (30 m^3^ ha^−1^) and by spraying the plants twice with the *Spirodela polyrhiza* extract (7 L ha^−1^) (Figure 1A,B and Figure 2A,B). In this synergistic system, the plants fertilized with ash, the digestate and *Spirodela polyrhiza* extract grew the fastest and gave a significantly higher yield of fresh and dry biomass compared to those fertilized with each fertilizer separately or with ash together with the digestate or with *Spirodela polyrhiza* (Figure 3A,B and Figure 4A,B). After fertilization with the newly developed mixture containing sorghum ash, the plants at the end of vegetation were 43% taller than the control, and they were 37% taller after the application of Jerusalem artichoke ash, while the yield of fresh biomass increased by 87 and 77%, respectively (Figure 1B, Figure 2B, Figure 3A and Figure 4A). 

### 2.3. Influence of Bio-Fertilizers on the Infection of Plants with Pathogenic Fungi

Plant infection with pathogenic fungi was reduced by the applied ash from burnt sorghum and Jerusalem artichoke, used alone or supplemented with the digestate and the *Spirodela polyrhiza* extract. As shown in Table 3, seven species of pathogenic fungi were isolated from the infected sorghum leaves, including those most dangerous for sorghum crops: *Colletotrichum* ssp., *Cercospora sorghi*, *Fusarium* spp., *Puccinia sorghi*, and *Sclerospora sorghi*. The presented data demonstrate that the percentage of all the isolated pathogenic fungi in relation to the total isolates and the percentage of infected plants were significantly decreased by all the fertilizers used alone in comparison to the control. The greatest decrease in the infection with pathogenic fungi was observed after the use of the sorghum and Jerusalem artichoke ash, in doses of 48 q ha^−1^, together with the digestate and the *Spirodela polyrhiza* extract. The plants treated with all these bio-fertilizers acting synergistically were significantly healthier than those treated with those fertilizers separately. However, only 4.8–4.9% of all the fertilized plants were infected by pathogens compared with 12.6% in the control (Table 3).

### 2.4. Effect of Bio-Fertilizers on Plant Physiological Activity

The presented advantageous changes in plant growth kinetics and health (Figure 1A and Figure 2A, Table 3) and biomass yield (Figure 3 and Figure 4), resulting from the applied fertilization treatments, were directly related to the improved activity of gas exchange and the increased index of chlorophyll content in leaves (Table 4). After the application of the two kinds of ash in combination with the digestate and the *Spirodela polyrhiza* extract, used in synergistic configurations promoting growth, the index of chlorophyll content in leaves increased proportionally to the plant growth and biomass yield, and it was 35% higher than in the control. Correspondingly, increased net photosynthesis, transpiration, stomatal conductance and decreased intercellular CO_2_, inversely proportional to the mentioned gas exchange parameters, were also observed. As in the case of growth kinetics (Figure 1A and Figure 2A), the sorghum ash also had a slightly more favorable effect on gas exchange and chlorophyll content in leaves than the ash from Jerusalem artichoke (Table 4). The combined application of all fertilizers resulted in the highest gas exchange activity and chlorophyll content compared to separate treatments. Simultaneously, this physiological activity assessed in all the treatment variants was always higher than in the control (Table 4). After fertilization with the prepared fertilizer mixture, the net photosynthesis, transpiration and stomatal conductance were 55–59, 90–96 and 77–91% higher, respectively, than in the control group. The higher values of these parameters were related to the use of the bio-fertilizer mixtures containing sorghum ash (Table 4).

The tested kinds of ash increased the activities of acid and alkaline phosphatase, RNase and dehydrogenases, proportionally to plant growth and the applied growth-promoting bio-fertilizers (Figure 1A and Figure 2A, Table 5). However, the sorghum ash was also slightly more effective than the ash from Jerusalem artichoke combustion. As presented above, the supplementation of ash with the digestate and the *Spirodela polyrhiza* extract additionally increased the activities of the assessed enzymes. These activities were also much higher when all the bio-fertilizers were applied together than when they were used separately (Table 5). Under the influence of fertilization with the developed mixture of bio-fertilizers, the activity of the assessed enzymes was higher by 40–68% than in the control. After the application of sorghum ash, this activity was about 6% higher than in the case of Jerusalem artichoke dust (Table 5). 

### 2.5. Effect of Bio-Fertilizers on Element Content in Leaves and Biomass Energy Properties

All three bio-fertilizers used together and acting synergistically had a very small effect on the amounts of macro- and microelements in sorghum leaves (Table 6) in spite of their significant positive influence on increasing the growth, health and physiological activity of the plants (Figure 1A and Figure 2A, Table 3, Table 4 and Table 5). The concentrations of elements (N, P, K, Mg, Fe, Mn) in the leaves of plants fertilized with the ash from the burnt sorghum and Jerusalem artichoke biomass and combined with the digestate and *Spirodela polyrhiza* foliar spray were 1–4% higher than in the control (Table 6). This combined application also increased slightly the calorific value of the biomass in the working state and slightly decreased the ash content in sorghum leaves and shoots. The values of the other measured energy parameters of sorghum biomass did not change (Table 7).

## 3. Discussion

The presented research demonstrates a new approach and proposes an innovative technology for the cultivation of energy plants, including sorghum, which involves their effective fertilization with ash from sorghum (*Sorghum bicolor* L. Moench) or Jerusalem artichoke (*Helianthus tuberosus* L.) biomass, acting synergistically with a digestate from a biogas plant and a *Spirodela polyrhiza* extract. All of these bio-fertilizers are alternatives to artificial fertilizers and strongly stimulate the growth and health of plants, and they additionally sequester CO_2_ from the atmosphere by themselves or during their production [27]. It is especially important to stop climate change, considering that the balance of greenhouse gas emissions resulting from the use of energy plants in power production is more favorable than in the case of fossil fuels. Additionally, these plants capture significant amounts of atmospheric CO_2_ during their growth. Moreover, biogas plant digestate, which is an effective bio-fertilizer, is obtained from CO_2_-absorbing energy plants and can also by itself capture this gas from the air [17,19,24]. In turn, *Spirodela polyrhiza*, produced for fertilizing purposes, also absorbs significant amounts of atmospheric CO_2_ and is highly recommended as an ecological fertilizer because it contains high amounts of key elements and bioactive compounds for plant growth. As the results show, the combined fertilization with ash, digestate and *Spirodela polyrhiza* extract highly increases the productivity of sorghum plants and eliminates the need for artificial fertilizers and pesticides. The tested bio-fertilizers and more vigorously growing plants also enhance CO_2_ sequestration, ultimately leading to a reduction in this gas in the atmosphere. Moreover, such fertilization enables a closed circulation of nutrients between plant and soil, maintaining high soil fertility [7].

This paper proposes the use of biomass ash to fertilize energy crops in a circular closed system, and it also solves the economically important problem of ash storage in landfills, which is expensive, dangerous to the environment and leads to the loss of valuable resources [30,31]. The study resulted in the obtaining of novel information concerning the differences and similarities in the nutrient composition and fertilizing efficiency of ash from sorghum and Jerusalem artichoke biomass. The differences found depended on the species of burnt plants, even though they were cultivated in the same field for only one vegetative season and were similarly fertilized. The fertilizing dust, irrespective of its origin, contained a limited amount of nitrogen because the majority of it was released to the atmosphere during the combustion of the biomass. 

The differences found in the amounts of macroelements in the two kinds of ash also remained in similar relations in the fertilized soil. The application of the sorghum ash to the podzolic soil resulted in a greater increase in the total amount of most macro- and microelements, and most importantly, it increased the available N.NO_3_, P, K and Mg. This resulted in its having a greater fertilizing efficiency in comparison with the Jerusalem artichoke ash. However, the pH was 12 regardless of the origin of the ash, which is similar to what is found in ash from other crops [9,16,32,33]. The problem of an excessively high pH could become serious in the case of alkaline soils. However, this is not a major problem in many countries; for example, in several regions of Poland, even 40–60% of soils are acidified [28,34]. In the present study, the pH of the podzolic soil was only 5.0; thus, increasing it up to 5.8–6.0 by applying ash was not dangerous and even favorable to the crop and the uptake of elements by the plants [35]. The low alkaline reaction in the soil could also be caused by the relatively low ash doses, high sorption capacity of the soil and its large volume compared to the amount of dust applied to it. The greater fertilizing usefulness of sorghum ash could also result from the fact that its weight per unit volume was half that of the ash from Jerusalem artichoke. Hence, a larger volume of the same weight added to the soil could have a more favorable effect on its structure, but no studies have been carried out in this respect. The higher concentration of elements in the sorghum ash resulted in their increased amount in the soil, which had a more beneficial effect on plant growth and metabolic processes than after the use of Jerusalem artichoke ash. Moreover, the increase in pH from 5.0 up to 5.8–6.0 could affect the greater uptake of elements by plants. These findings are in line with those of the studies by Schiemenz et al. [9] and Zając et al. [8], which indicated great differences in elemental structure of dusts depending on the burnt plants and also on the content in them of organic and inorganic substances, possible impurities, the sampling point, the weather during the vegetative season, the harvesting conditions, the combustion method, and various other factors affecting plant growth [5,6]. Thus, the ash should be analyzed before its use for fertilizing purposes [9]. The fertilizing and health-promoting efficiency of the tested biomass dust is also in line with the research by Naghipour et al. [36] showing an improved germination of *Bromus tomentellus* and *Bromus tectorum* seeds after treatment with ash. However, in the other species studied by them, no response or inhibiting reactions were observed under the influence of this ash. These results are also consistent with the studies by Meller and Bilenda [10], showing an increase in the amount of available potassium in the soil from medium to very high after fertilization with ash from straw, maize, energy willow, and wood chips. They also established that the physicochemical properties of soils after enrichment with this ash were comparable to those obtained with mineral fertilizers, and they were even more favorable in some cases. Similarly, Schiemenz and Eichler-Löbermann [9] showed that the amount of phosphorus in the ash from burnt crops can be similar to that in a commercial fertilizer. These data are very important from the point of view of improving the quality of the agrological environment by the use of ash instead of synthetic fertilizers. The demonstrated research precisely specifies the fertilization options and differences in element composition of ash originating from different annul crops, and it confirms the findings by Zając et al. [8] that ash from annual crops may contain no heavy metals. However, the literature concerning the fertilizing efficiency of ash from annual plants, presumably free of toxic substances, is still limited. Most of it concerns wood ash, which may contain higher levels of As, Pb, Ni and Cr, or Cu, Mn and Zn [16]. This is mainly due to a longer period of tree growth, sometimes in a polluted area, which are both conducive to a greater accumulation of these metals, in contrast to annual crops cultivated for six months only or shorter in unpolluted areas [37]. Smołka-Danielowska and Jabłońska [11] demonstrate the high variability of potentially toxic elements in wood biomass ash, whose concentrations exceeded the values typical for these types of biomass specified in the Polish standard. Thus, this dust cannot be used for fertilizing crops. However, in spite of the possibility of pollutants being present in woody ash, a great number of literature positions demonstrate a positive impact of it on the growth of several plant species and recommend it for use as a plant fertilizer [13,38]. In contrast to those studies, our research, and that of Zając at al. [8], indicated that ash from Jerusalem artichoke and sorghum, and also from annual cereals, does not contain heavy metals, which indicates the possibility of using it in agriculture to replace artificial fertilizers in order to improve the natural environment. Moreover, such ash contains CaO, which binds CO_2_ from the air and has strong antimicrobial properties, so it prevents plant diseases, replacing the need to use pesticides that pollute the environment [39]. An additional advantage of using this kind of ash in agriculture is its positive effect on the growth of aquatic plants (*Lemnaceae*), and therefore it is also friendly to the aquatic environment, as our previous research had shown.

The presented research indicates the possibility of increasing the beneficial effect of the tested ash on soil fertility and on the growth and physiological parameters of sorghum plants by additionally using a digestate from a biogas plant and a *Spirodela polyrhiza* extract in newly developed synergistic configurations and doses. The obtained synergism of ash, digestate and *Spirodela polyrhiza* extract allowed fertilization of the soil with the tested ash at relatively the most effective dose to prevent an excessive increase in pH, knowing that the pH of the ash was 12.0. The obtained synergistic effect raised the plant growth rate and the studied physiological activities to a greater extent than each of the mentioned fertilizers used separately. The high efficiency of the described novel methods allowed a reduction in the occurrence of toxic substances in the soil, due to the possibility of lowering ash doses, and in the use of pesticides and artificial fertilizers applied to crops. The obtained results permit us to regard these biomass combustion wastes as new valuable fertilizers in sustainable, circular agriculture, which are environmentally friendly and useful as a soil improvement component with additional CO_2_ capture properties [40]. The positive synergistic impact of ash, biogas plant digestate and *Spirodela polyrhiza* extract on plant development is most likely caused by the additional amounts of macro- and microelements contained in these bio-fertilizers and also through their interactions with one another, including ash or digestate increasing the development of the tested aquatic plants [26]. Moreover, not only does the biogas plant digestate contain nutrients, but it also harbors microorganisms that could further support soil fertility and element uptake by plants [41].

The high fertilizing value of *Spirodela polyrhiza* was evidenced by the amount of macro- and microelements in its biomass, which was lower than in the ash and much higher than in the soil. These elements evidently had a crucial role in the improvement of sorghum plant growth, similar to the extract from aquatic *Lemna minor* (*Lemnaceae* family), which in a concentration of 0.05–1.00% stimulated the assimilation of nitrogen, phosphorous, potassium, calcium, magnesium, sodium, iron, and copper [29]. Another element that greatly increases the fertilizing efficiency of *Spirodela polyrhiza* is the extremely high boron content in its biomass in relation to that in ash and soil. This important micronutrient takes part in the biochemical, physiological, and morphological development of plants. Boron promotes root development, raises plant pigment content, reduces the number of empty grains, and improves fruit and seed setting. Moreover, it increases the uptake and translocation of micro- and macronutrients (P, N, K, Zn, Fe, and Cu) in plants. Therefore, increased amounts of boron and other growth-stimulating compounds positively influencing the kernel germination, biomass yield, leaf area, and vigor are most likely to be of special importance in the synergistic effect of *Spirodela polyrhisa*, ash and digestate on the development of sorghum plants [42]. In view of this metabolism, this aquatic plant can enhance the fertilizing efficiency of the tested ash and enables the use of it in lower doses, thus helping to avoid an excessive increase in soil pH. The significant impact of *Spirodela polyrhiza* on increasing the fertilizing efficiency of ash could also be a result of its ability to sequester CO_2_ during the photosynthesis process [43] and the excessive accumulation of nutrients from water environment, also processing them into valuable bioproducts. This activity was also found by Buono et al. [29] and Pulido et al. [44] studying the impact of duckweed (*Lemna minor* L.), belonging to the same *Lemnaceae* family, on vegetable plant development. The applied duckweed helped to obtain yields of beetroot (*Beta vulgaris* L.), tomato (*Solanum lycopersicum* L.), and sorghum (*Sorghum bicolor* L.) comparable to those produced with non-organic fertilizers. Thus, duckweed, including *Spirodela polyrhiza*, may be used as a substitute for artificial fertilizers without a negative impact on the yield and quality of food [29,44].

The tested kinds of ash, preferably acting synergistically with the digestate and the *Spirodela polyrhiza* extract, are environmentally friendly, since apart from their biological fertilizing properties, they also decrease the infection of sorghum plants with pathogenic fungi, which are dangerous to plants, as was evident in the presented and previous research by the authors [7]. Therefore, they can be used for protecting plants against pathogens as an alternative to pesticides [39,45]. This effect could be caused by the biocidal properties of ash and the higher resistance of the more vigorous sorghum to infection by pathogenic microflora and also by the fungicidal properties of *Spirodela polyrhiza*, as was found after fertilizing corn and willow with a digestate from corn grain biodigestion to methane or treatment with *Cyanobacteria* [22]. The applied bio-fertilizers could furthermore directly stimulate plants to produce antioxidants and other compounds that inhibit pathogen development by regulating lignin and suberin biosynthesis in cell walls. Increasing the stiffness and impermeability of these walls hinders the penetration of tissues by pathogens and the process of infection [45]. The obtained results are in line with the study by Oguntade and Adedotun [46] showing that the seeds of corn (*Zea mays* L.), melon (*Cucumis melo* L.) and bean (*Phaseolus vulgaris* L.) stored with ash from *Nauclea diderrichii* and *Piptadeniastrum africanum* were the healthiest, with less fungal growth on them and elimination of weevils, compared to the seeds stored with the fungicide Benlate, which was only effective against fungal growth.

The increased growth and yield of biomass, caused by the applied ash enriched with the digestate and the *Spirodela polyrhiza* extract, were associated with the proportional favorable changes in plant physiological activity, as assessed by the index of chlorophyll content in leaves, gas exchange (net photosynthesis, transpiration, stomatal conductance, and intercellular CO_2_ concentration) and enzyme activity (alkaline and acid phosphatase, RNase, total dehydrogenase), which play a key role in plant development and are recommended as markers of plant development [22,32,47,48]. This beneficial result indicates a positive reaction of the plant metabolic chain to ash, digestate and *Spirodela polyrhiza*, and it shows that their synergistic effect was the most effective in intensifying these metabolic processes. The relationship between plant growth and physiological processes and their dependence on the degree of tissue enrichment with nutrients is well described in the literature. Among others, Kalaji et al. [49] demonstrated that the nutrient content in plants determined the intensity of photochemical photosynthesis and chlorophyll fluorescence. A deficit in the amounts of these elements slowed these physiological processes, which in turn negatively affected plant growth. The close relationship between the degree of sorghum enrichment with nutrients present in different doses of biogas plant digestate and metabolic activity, as well as the impact of this relationship on plant development, had also been described and justified by Romanowska-Duda et al. [22,50]. 

Another environmentally friendly result of the tested treatments is the slight increase in the concentration of the assessed macroelements in leaves. The outcome is in line with the research by Zapałowska et al. [51], who stated that fertilization with biomass ash from coniferous trees increased the amount of K in leaves, whose amount grew by 30%. The present study also showed that the use of ash, digestate and *Spirodela polyrhiza* extract, in addition to increasing the biomass of sorghum, slightly increased its calorific value in the working state and slightly reduced the ash content in its biomass. This may be associated with a slightly changed element content in plants under the influence of ash fertilization, which determines the amount of energy compounds in tissues. The slight modifications of both parameters indicated that the quality of sorghum was not significantly changed by the applied bio-fertilizers. Therefore, these plants can be used for silage for animal nutrition and energy purposes. Fertilizing them in accordance with the developed recipe reduces the need to use toxic artificial fertilizers. *Spirodela polyrhiza* also has a high ability to store starch under unfavorable growth conditions, which allows this plant to be used in the production of biofuels [52].

**Figure 5:**
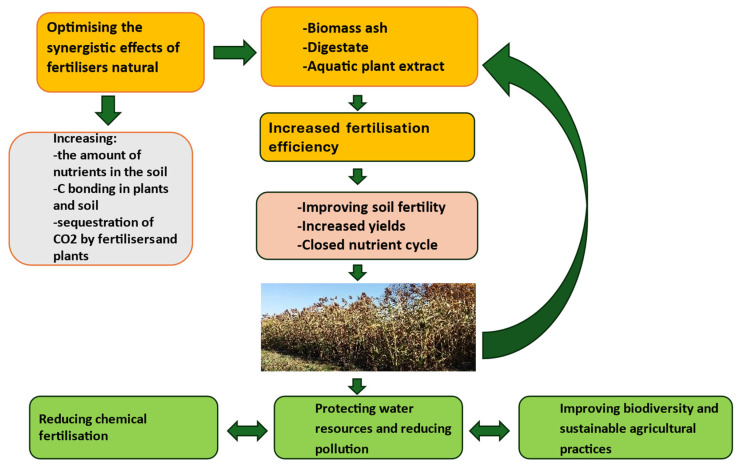
Closed circulation of elements and increased soil fertility and productivity of sorghum plants grown in a closed system, achieved by the synergistic effect of biomass ash, digestate and *Spirodela polyrhiza* extract, which, by additionally sequestering CO_2_ and reducing chemical fertilization and pesticide needs, improve the quality of the environment.

The performed research indicates the possibility of building a model of cultivating energy crops in a circular system, in which nutrients, as a result of using the newly developed technology, move in a closed circuit between plant and soil and are not released from a given agro-technical environment, which results in increased soil fertility [5,7,8,22,52,53]. Enriching the ash with the digestate and the *Spirodela polyrhiza* extract additionally increases the pool of nutrients and other plant growth stimulants and also the intensity of CO_2_ sequestration not only on land but also in the aquatic environment. Therefore, the developed technology helps not only to increase the yield of sorghum biomass but also to improve the quality of the land and water environment, as shown in Figure 5. However, long-term, intensive research or test programs are still necessary, which will include constant monitoring and examination of the proposed technology under various environmental conditions [54].

## 4. Material and Methods 

### 4.1. Plants, Ash, Digestate and Soil 

The sorghum grains (*Sorghum bicolor* L. Moench) ‘Rona 1’ were supplied by the breeding company “Kutnowska Hodowla Buraka Cukrowego” in Poland. Sorghum is beginning to be cultivated on a large scale in central Europe as an alternative to maize, and it requires the development of innovative, environmentally friendly cultivation technologies [55].

The ash had been acquired by burning dry biomass of sorghum (*Sorghum bicolor* L. Moench) ‘Rona 1’ and Jerusalem artichoke (*Helianthus tuberosus* L.) plants as part of our own previous experiments. It was sifted through sieves (with 2 × 2 mm mesh) before application. The proposed dosages of the ash (48 q ha^−1^) had been elaborated on in several preliminary research works, which are both unpublished and published [7].

The liquid non-centrifuged digestate from corn grain biodigestion to methane was supplied by the distillery integrated with the biogas plant in Piaszczyna (Gamawind sp. z o.o., Poznań, Poland), which produces alcohol and biogas. The applied dose was selected on the basis of previous studies [22].

Plants of *Spirodela polyrhiza* (*Spirodela polyrhiza* L. Schleid), belonging to the *Lemnaceae* family, were isolated from a water reservoir in Lodz voivodeship (Poland) (19°28′ E, 51°45′ N). The plants were grown in in vitro cultures in tap water supplemented with 2.5% of the digestate from the biogas plant and ozonized for 12 min. The plans were cultivated in a phytotron at 24 °C for 14 days with continuous lighting supplied by 2 × 18 W/840 Philips Master LT-D lamps (Amsterdam, The Netherlands) [26]. To prepare an aqueous extract from the cultured in vitro plants, 10 g of fresh *Spirodela polyrhiza* biomass was placed in an extraction vessel with 100 mL of tap water. The maceration process was conducted at room temperature (23–24 °C) in a mixer to obtain a uniform homogenate (for 5 min). To assess the fertilizing properties, the obtained homogenate (extract) was applied to the plants in the form of a spray in a dose of 7 L ha^−1^ diluted in 300 liters of tap water [26,50]. The extract dose was chosen on the basis of our own previous experiments.

The soil used for plant cultivation in the field was classified as podzolic. The compositions of the soil, the two kinds of ash, the digestate from the biogas plant and of the *Spirodela polyrhiza* extract are presented in Table 1 and Table 2.

### 4.2. Treatment of Soil and Plants with Ash, Digestate and Spirodela Polyrhiza Extract

The research was performed in northern Poland in a field (54°01′21″ N, 17°10′19″ E) located 80 km from the Baltic Sea. The temperature in this area oscillated between 11 and 21 °C, precipitation was 655 mm, and the air was humid from the nearby Baltic Sea. 

Before seed sowing, at the end of April, the podzolic soil in designated plots (experimental variants), 3 × 3 m in size, were fertilized with sorghum ash (48 q ha^−1^) or Jerusalem artichoke ash (48 q ha^−1^), and then the selected plots were fertilized with digestate (30 m^3^ ha^−1^). Next, the soil was loosened to a depth of 15 cm in order to mix it uniformly with the applied bio-fertilizers. At the beginning and in the middle of June (at a two-week interval), the plants grown on the designated plots were additionally sprayed with the *Spirodela polyrhiza* extract (7 L ha^−1^). The experimental variants are presented in Figure 1, Figure 2, Figure 3 and Figure 4 and Table 1, Table 2, Table 3, Table 4, Table 5, Table 6 and Table 7. The tested bio-fertilizers were applied as follows:

1. Sorghum ash (48 q ha^−1^) to soil;
2. Sorghum ash (48 q ha^−1^) and digestate 30 m^3^ ha^−1^ to soil;
3. Sorghum ash (48 q ha^−1^) to soil and two-time foliar application of *Spirodela polyrhiza* extract (7 L ha^−1^);
4. Sorghum ash (48 q ha^−1^) and digestate 30 m^3^ ha^−1^ to soil and two-time foliar application of *Spirodela polyrhiza* extract (7 L ha^−1^);
5. Jerusalem artichoke ash (48 q ha^−1^) to soil;
6. Jerusalem artichoke ash (48 q ha^−1^) and digestate 30 m^3^ ha^−1^ to soil;
7. Jerusalem artichoke ash (48 q ha^−1^) to soil and two-time foliar application of *Spirodela polyrhiza* extract (7 L ha^−1^);
8. Jerusalem artichoke ash (48 q ha^−1^) and digestate 30 m^3^ ha^−1^ to soil and two-time foliar application of *Spirodela polyrhiza* extract (7 L ha^−1^).

The fertilized field plots were situated randomly, and each experimental variant was performed in three repetitions. 

In the middle of May, the sorghum grains were sown into the soil in all the field plots fertilized previously as described above. The grains, 175 per field plot, were sown in rows 35 cm apart, and the distance between the seeds in a row was 12 cm, as in large-scale production. 

Non-fertilized plants and plots were the control. The treatments of soil with the digestate alone and the two-time foliar application of *Spirodela polyrhiza* extract to plants on its own were additional controls used to check the impact of these bio-fertilizers on plant development.

### 4.3. Assessments of Soil and the Development, Health and Physiological Activity of Plants 

The effects of the applied fertilization methods on soil properties and plant development were assessed on the basis of nutrient content in the soil, plant growth dynamics and biomass yield. They were also assessed by determining plant infection with pathogenic microflora, gas exchange, index of chlorophyll content, activity of selected enzymes and element content, which were carried out on fully developed leaves situated below the top of plants. In each experimental variant, one leaf from each of the fifty plants or 100 g of them were taken in the morning hours of sunny days in July for the assessment of nutrient content. 

The kinetics of sorghum shoot growth were measured every month with a ruler from the soil surface up to the top of plant [22,56]. 

Fresh (freshly harvested) and dry biomass weights (after 3-day drying at 130 °C in a hot-air oven) of ten whole plants per experimental variant were determined at the end of the growing season and converted to weight per one plant [22].

The activity of gas exchange (net photosynthesis, transpiration, stomatal conductance, intercellular CO_2_ concentration) was measured using the TPS-2 portable photosynthesis measurement system (PP Systems, Amesbury, MA 01913, USA) [49].

The index of chlorophyll content (in SPAD units) in leaves was measured with a Minolta SPAD-502 chlorophyll meter (Konica Minolta Inc., Tokyo, Japan) [22,56].

The diagnosis of pathogenic fungi consisted of placing fragments of infected sorghum leaves in moist cameras or on diagnostic media specific for individual types of pathogens, containing antibiotics that inhibit bacterial growth. The cultures were incubated until fungal spores were formed. Direct isolation was then performed, and spore cultures derived from single spores were grown according to the methods described by Gams et al. [57] and Waller et al. [58]. Fungi were identified after 4–6 days by means of morphological, morphological and developmental, and morphological and ecological criteria, using diagnostic keys allowing their affiliation to different taxa ranks [58,59,60]. The names and classification of fungi were determined in accordance with the regulations established by the International Code of Nomenclature for algae, fungi, and plants [61]. The percentage of plants infected with pathogenic fungi was determined using 175 plants on each plot. 

The activities of alkaline (EC 3.1.3.1) and acid (EC 3.1.3.2) phosphatase and RNase (EC 3.1.27.5) in leaves were studied by the methods developed by Knypl and Kabzińska [62]. 

Total dehydrogenase activity (EC 1.1.1.-) was assessed using the technique presented by Romanowska-Duda et al. [22]. A UV mini-1240 Shimadzu spectrophotometer (Shimadzu Scientific Instruments, Kyoto, Japan) was used for formazan determination at a wavelength of 480 nm. Activities of the total dehydrogenases were calculated on the basis of fresh mass (fm).

The concentrations of macro- and microelements in the ash from burnt sorghum and Jerusalem artichoke biomass, in the digestate from the biogas plant and in the *Spirodela polyrhiza* extract, as well as in the podzolic soil before and after fertilization with the ash, digestate and *Spirodela polyrhiza* extract, and in sorghum leaves, were determined in a certified laboratory of The Institute of Horticulture–National Research Institute in Skierniewice, Lodz voivodeship (Poland), using standard procedures. After the evaporation of excess water, the concentrated material was mineralized in an “Etho-1” microwave oven (Milestone, Sorisole (BG), Italy). Then, the quantity and composition of elements were determined using a plasma spectrometer (ICP) model DV2000 (PerkinElmer Inc., Alexandria, VA 22309, USA) at different spectral wavelengths. The total nitrogen content was determined by the Kjeldahl method carried out in three stages: (i) mineralization of the sample in the presence of a suitable catalyst in a boiling mixture of sulfuric acid, (ii) alkalization of the solution to release ammonia, and (iii) distillation of the released ammonia with steam. The nitrogen content was determined by titration [63]. The energetic value of plants was estimated by Carbochem, a certified laboratory in Sosnowiec, Silesian voivodeship (Poland), using Polish assessment standards [22,64,65,66].

### 4.4. Statistical Analysis 

The presented experiments were conducted in three series and in three replicates for each experimental variant. Each repetition, within each series, was set up in a completely randomized block design. The obtained data, given as means from replicates and series, were processed by applying analysis of variance (ANOVA I) in Statistica 12. The data presented in tables and figures were grouped employing the Newman–Keuls multiple range test at the α = 0.05 significance level.

## 5. Conclusions

The rational management of greatly increasing amounts of biomass ash in sustainable agriculture and improvement of its fertilizing efficiency have become some of the most important issues in the global economy, taking into account environment protection, bioenergy, and low emissions and sequestration of CO_2_. The presented research demonstrates a new approach and proposes an innovative technology for the cultivation of energy plants, involving ecological fertilization with ash from sorghum (*Sorghum bicolor* L. Moench) and Jerusalem artichoke (*Helianthus tuberosus* L.) biomass, acting synergistically with a biogas plant digestate and a *Spirodela polyrhiza* extract, which have CO_2_ sequestration ability. The presented results show the differences in the physical properties, nutrient composition and fertilizing efficiency of the ash from sorghum and Jerusalem artichoke when used separately in optimized doses. They also indicate the possibility of increasing the beneficial effect of ash on soil fertility and the growth and physiological properties of sorghum plants by using an environmentally friendly biogas plant digestate and a *Spirodela polyrhiza* extract in a newly developed synergistic formula. The tested ash, applied in doses of 48 q ha^−1^, alone, or best enriched with the digestate and the *Spirodela polyrhiza* extract, increased the concentration of N, P, K and Mg in the podzolic soil by 20, 15, 4 and 14%, respectively. After fertilization with the newly developed mixture containing sorghum ash, the plants were 43% taller than in the control, and after the application of Jerusalem artichoke dust by 37%, while the yield of fresh biomass increased by 87 and 77%, respectively. Plants fertilized in this way were less infected with pathogens and showed twice as high photosynthetic activity as in the control. The use of the synergistically acting bio-fertilizers had a very small effect on the amounts of macro- and microelements in sorghum leaves. The ash from sorghum biomass was slightly more efficient than that from Jerusalem artichoke. The beneficial effects on plants of the tested ash used together with the digestate and the *Spirodela polyrhiza* extract indicate that this combination of bio-fertilizers can help reduce the use of synthetic fertilizers and pesticides. At the same time, the possibility of CO_2_ sequestration by these bio-fertilizers and intensively growing plants under their influence have a beneficial effect on the environment. Therefore, this technology, involving fertilization with synergistically acting ash from sorghum or Jerusalem artichoke, a digestate and a *Spirodela polyrhiza* extract, can be proposed for the cultivation of energy plants in circular, sustainable agriculture.

## Figures and Tables

**Table 1:** Concentrations of elements in the sorghum (aS) and Jerusalem artichoke (aJ) ash, the biogas plant digestate, *Spirodela polyrhiza* biomass, and the soil before and after fertilization with sorghum (aS) or Jerusalem artichoke (aJ) ash (48 q ha^−1^) together with the biogas plant digestate (D; 30 m^3^ ha^−1^).

Assessed Materials	N	C^a^	P	K	Ca	Mg	Fe	Mn	Cu	Zn	B	Dry Mass
	%	[mg kg^−1^ dry weight]										[%]
aJ	0.40 ^b^*	34,021 ^a^	13,461 ^b^	155,202 ^b^	138,436 ^b^	10,099 ^b^	2452 ^a^	155 ^a^	29.1 ^b^	856 ^b^	72 ^b^	92.9 ^a^
aS	0.50 ^a^	34,204 ^a^	13,990 ^a^	162,897 ^a^	43,800 ^a^	11,690 ^a^	2229 ^a^	158 ^a^	30.7 ^b^	1040 ^a^	92 ^b^	91.6 ^b^
Soil not fertilized	0.09 ^d^	-	951 ^f^	3831 ^f^	2945 ^f^	1602 ^f^	659 ^c^	46.1 ^e^	23.9 ^c^	29.8 ^e^	62.8 ^c^	50.0 ^c^
Soil fertilized aJ + D	0.10 ^c^	-	1053 ^e^	4928 ^e^	32,878 ^d^	2245 ^e^	988 ^b^	70.1 ^d^	24.9 ^c^	35.8 ^de^	65.3 ^c^	50.2 ^c^
Soil fertilized aS + D	0.12 ^b^	-	1220 ^d^	5129 ^d^	30,562 ^c^	2573 ^d^	995 ^b^	72.5 ^c^	25.9 ^c^	36.2 ^d^	63.9 ^c^	50.3 ^c^
*Spirodela polyrhiza*	-	-	7288 ^c^	45,232 ^c^	17,936 ^e^	3468 ^c^	496 ^d^	153 ^b^	55.4 ^a^	143 ^c^	448 ^a^	- -
LSD_0.05_	0.009	350.6	102.8	201.9	450.3	230.2	125	13.1	2.3	5.9	10.4	0.3
		[mg L^−1^]										
Digestate	2455 ^a^	-	269 ^a^	992 ^a^	298 ^a^	117 ^a^	9.0 ^a^	0.330 ^a^	0.182 ^a^	0.982 ^a^	3.371 ^a^	1.4 ^a^
LSD_0.05_	1.2		1.1	1.0	1.4	1.5	1.1	0.1	0.05	0.2	0.2	0.3

C^a^ unburnt carbon, pH of ash—12.0, pH of biogas plant digestate—7.6. Ash content in sorghum biomass—3.88%, in J. artichoke—3.78%. * The data marked with the same letters within a column and measurement units ([mg kg^−1^ dry weight] or [mg L^−1^]) are not significantly different, according to Newman–Keuls multiple range test at an α level of 0.05.

**Table 2:** Concentrations of available nutrients in the soil before and after fertilization with 48 q ha^−1^ of ash from Jerusalem artichoke (aJ) or sorghum (aS) biomass applied to the soil together with the biogas plant digestate (D; 30 m^3^ ha^−1^).

Soil Fertilization	pH in H_2_O	Salinity g NaCl L^−1^	C^a^	N.NO_3_	P	K	Mg	Ca
			%	mg L^−1^ Soil				
Soil not fertilized	5.0 ^b^*	0.25 ^a^	1.20 ^b^	118 ^b^	135 ^b^	227 ^c^	91.0 ^c^	1030 ^c^
Soil + aJ + D	6.0 ^a^	0.25 ^a^	1.21 ^a^	139 ^a^	156 ^a^	254 ^b^	102 ^b^	2204 ^a^
Soil + aS + D	5.8 ^a^	0.25 ^a^	1.22 ^a^	144 ^a^	160 ^a^	261 ^a^	110 ^a^	2099 ^b^
LSD_0.05_	0.3	0.02	0.03	6.9	5.1	6.2	7.0	104.2

C^a^—organic carbon content, humus content in soil—2.01%, humus layer—20 cm. * The data marked with the same letters within a column are not significantly different, according to the Newman–Keuls multiple range test at an α level of 0.05.

**Table 3:** Percentage of pathogenic fungi species on the infected leaves in relation to the total isolates and the percentage of infected sorghum plants fertilized with the ash from burnt sorghum (aS) or Jerusalem artichoke (aJ) in doses of 48 q ha^−1^, applied to the soil alone or together with the biogas plant digestate (D; 30 m^3^ ha^−1^) and/or treated with the *Spirodela polyrhiza* extract (Sp; 7 L ha^−1^) as foliar spray.

Plant Fertilization	Pathogenic Fungi							Percentage of Infected Plants
	*Colleto-trichum* ssp.	*Cercospora sorghi*	*Fusarium* spp.	*Puccinia sorghi*	*Sclerospora sorghi*	*Pythium* spp.	*Rhizoctonia* spp.	
Control	7.5 ^a^*	3.9 ^a^	3.0 ^a^	3.0 ^a^	4.9 ^a^	1.6 ^a^	1.5 ^a^	17.8 ^a^
D	6.0 ^c^	3.5 ^c^	1.5 ^c^	2.3 ^c^	3.0 ^c^	0.9 ^c^	1.2 ^c^	11.2 ^c^
Sp	6.5 ^b^	3.7 ^b^	1.8 ^b^	2.5 ^b^	3.5 ^b^	1.2 ^b^	1.3 ^b^	12.6 ^b^
Fertilization with sorghum ash, digestate and *Spirodela polyrhiza*								
aS 48	5.5 ^d^	3.3 ^d^	1.2 ^d^	2.0 ^d^	2.7 ^d^	0.7 ^d^	1.0 ^d^	10.5 ^d^
aS48 + D	4.2 ^g^	2.1 ^h^	0.5 ^f^	1.3 ^f^	2.1 ^f^	0.3 ^f^	0.8 ^e^	5.6 ^h^
aS48 + Sp	4.2 ^g^	2.3 ^g^	0.0 ^g^	0.5 ^g^	2.4 ^e^	0.0 ^g^	0.7 ^f^	7.0 ^g^
aS48 + D + Sp	4.0 ^h^	1.1 ^i^	0.0 ^g^	0.0 ^h^	1.7 ^g^	0.0 ^g^	0.0 ^g^	4.8 ^i^
Fertilization with Jerusalem artichoke ash, digestate and *Spirodela polyrhiza*								
aJ48	5.5 ^d^	3.3 ^d^	1.2 ^d^	2.0 ^d^	2.7 ^d^	0.7 ^d^	1.0 ^d^	10.5 ^d^
aJ48 + D	4.4 ^f^	2.5 ^f^	0.5 ^f^	1.3 ^f^	2.1 ^f^	0.3 ^f^	1.0 ^d^	7.3 ^f^
aJ48 + Sp	4.6 ^e^	3.0 ^e^	0.9 ^e^	1.8 ^e^	2.1 ^f^	0.5 ^e^	1.0 ^d^	7.5 ^e^
aJ48 + D + Sp	4.0 ^h^	1.2 ^i^	0.0 ^g^	0.0 ^h^	1.7 ^g^	0.0 ^g^	0.0 ^g^	4.9 ^i^
LSD_0.05_	0.15	0.15	0.20	0.18	0.25	0.10	0.10	0.5

* The data marked with the same letters within a column are not significantly different, according to Newman–Keuls multiple range test at an α level of 0.05.

**Table 4:** Gas exchange and index of chlorophyll content in the leaves of sorghum plants fertilized with ash from burnt sorghum (aS) or Jerusalem artichoke (aJ) biomass applied to the soil alone (in doses of 48 q ha^−1^) or together with the biogas plant digestate (D; 30 m^3^ ha^−1^) and/or treated with the *Spirodela polyrhiza* extract (Sp; 7 L ha^−1^) as foliar spray.

Plant Fertilization	Net Photosynthesis [µm CO_2_ m^−2^ s^−1^]	Transpiration [mmol H_2_O m^−2^ s^−1^]	Stomatal Conductance [mmol H_2_O^−1^ M^−2^ s^−1^]	Intercellular CO_2_ Concentration [µmol CO_2_ Air mol^−1^]	Index of Chlorophyll Content [SPAD]
Control	4.80 ^j^*	0.85 ^i^	153.0 ^j^	309.0 ^a^	20.4 ^f^
D	5.11 ^h^	1.08 ^g^	206.0 ^h^	280.0 ^c^	22.1 ^d^
Sp	4.89 ^i^	0.96 ^h^	187.0 ^i^	291.0 ^b^	21.2 ^e^
Fertilization with sorghum ash, digestate and *Spirodela polyrhiza*					
aS 48	5.70 ^f^	1.36 ^f^	252.0 ^f^	271.0 ^d^	23.1 ^f^
aS 48 + D	7.40 ^c^	1.72 ^c^	311.0 ^c^	241.0 ^g^	26.9 ^c^
aS 48 + Sp	7.20 ^d^	1.48 ^e^	274.0 ^e^	260.0 ^e^	24.2 ^e^
aS 48 + D + Sp	7.80 ^a^	1.98 ^a^	358.0 ^a^	212.0 ^i^	28.7 ^a^
Fertilization with Jerusalem artichoke ash, digestate and *Spirodela polyrhiza*					
aJ 48	5.30 ^g^	1.34 ^f^	234.0 ^g^	269.0 ^d^	23.0 ^f^
aJ 48 + D	7.40 ^c^	1.71 ^c^	312.0 ^c^	231.0 ^h^	26.3 ^c^
aJ 48 + Sp	7.00 ^e^	1.60 ^d^	289.0 ^d^	252.0 ^f^	27.7 ^b^
aJ 48 + D + Sp	7.60 ^b^	1.83 ^b^	331.0 ^b^	213.0 ^i^	28.6 ^a^
LSD_0.05_	0.18	0.10	16.2	7.4	0.7

* The data marked with the same letters within a column are not significantly different, according to Newman–Keuls multiple range test at an α level of 0.05.

**Table 5:** Activities of selected enzymes in the leaves of sorghum grown in soil fertilized with ash from burnt sorghum (aS) or Jerusalem artichoke (aJ) applied to the soil alone (in doses of 48 q ha^−1^) or together with the biogas plant digestate (D, 30 m^3^ ha^−1^) and/or treated with the *Spirodela polyrhiza* extract (Sp; 7 L ha^−1^) as foliar spray.

Plant Fertilization	Phosphatase (pH = 6.0) [U g^−1^ f.w.]	Phosphatase (pH = 7.5) [U g^−1^ f.w.]	RNase [U g^−1^ f.w.]	Total Dehydrogenases [mg Formazan g Leaf ^−1^]
Control	0.51 ^j^*	0.22 ^j^	2.5 ^j^	0.46 ^j^
D	0.55 ^h^	0.27 ^h^	2.9 ^h^	0.53 ^h^
Sp	0.54 ^i^	0.25 ^i^	2.7 ^i^	0.50 ^i^
Fertilization with sorghum ash, digestate and *Spirodela polyrhiza*				
aS 48	0.64 ^f^	0.31 ^f^	3.3 ^f^	0.60 ^f^
aS 48 + D	0.76 ^b^	0.38 ^c^	3.9 ^c^	0.69 ^c^
aS 48 + Sp	0.73 ^c^	0.36 ^d^	3.7 ^d^	0.66 ^d^
aS 48 + D + Sp	0.79 ^a^	0.42 ^a^	4.3 ^a^	0.74 ^a^
Fertilization with Jerusalem artichoke ash, digestate and *Spirodela polyrhiza*				
aJ 48	0.61 ^g^	0.29 ^g^	3.1 ^g^	0.57 ^g^
aJ 48 + D	0.71 ^d^	0.38 ^c^	3.9 ^c^	0.69 ^c^
aJ 48 + Sp	0.69 ^e^	0.34 ^e^	3.5 ^e^	0.63 ^e^
aJ 48 + D + Sp	0.76 ^b^	0.40 ^b^	4.1 ^b^	0.71 ^b^
LSD_0.05_	0.02	0.015	0.10	0.02

* The data marked with the same letters within a column are not significantly different, according to Newman–Keuls multiple range test at an α level of 0.05.

**Table 6:** Concentrations of macro- and micronutrients in the leaves of sorghum plants fertilized with ash from burnt sorghum (aS) or Jerusalem artichoke (aJ) plants applied to the soil (in doses of 48 q ha^−1^) together with the biogas plant digestate (D; 30 m^3^ ha^−1^) and treated with the *Spirodela polyrhiza* extract (Sp; 7 L ha^−1^) as foliar spray.

Fertilizer	N	C	P	K	Ca	Mg	Na	S.SO_4_	Fe	Mn	Cu	Zn	B
	%	[mg kg^−1^ d.w.]											
Control	2.14 ^b^*	471,040 ^a^	4655 ^b^	21,284 ^b^	7508 ^b^	2569 ^b^	128 ^a^	1490 ^a^	285 ^b^	20.0 ^b^	13.0 ^a^	32.0 ^a^	18.3 ^a^
aS48 + D + Sp	2.24 ^a^	471,250 ^a^	4708 ^a^	21,500 ^a^	8177 ^a^	2624 ^a^	129 ^a^	1493 ^a^	309 ^a^	21.8 ^a^	13.5 ^a^	32.7 ^a^	18.8 ^a^
aJ48 + D + Sp	2.20 ^a^	471,232 ^a^	4701 ^a^	21,509 ^a^	8170 ^a^	2620 ^a^	127 ^a^	1489 ^a^	300 ^a^	21.4 ^a^	13.1 ^a^	32.0 ^a^	18.5 ^a^
LSD_0.05_	0.05	310.5	30.2	201.6	488	36.2	5.1	6.3	12.4	1.0	0.7	0.9	0.4

* The data marked with the same letters within a column are not significantly different, according to Newman–Keuls multiple range test at an α level of 0.05.

**Table 7:** Energy value of the sorghum plants fertilized with the ash from burnt sorghum (aS) or Jerusalem artichoke (aJ) biomass applied to the soil (in doses of 48 q ha^−1^) together with the biogas plant digestate (D; 30 m^3^ ha^−1^), and treated with the *Spirodela polyrhiza* extract (Sp; 7 L ha^−1^) as foliar spray.

Evaluated Properties	Unit of Measure	Plants Not Fertilized Control	Plants Fertilized with:		LSD_0.05_
			aS 48 + D + Sp	aJ 48 + D + Sp	
	Analytical state				
Analytical humidity	[%]	5.11 ^a^*	5.30 ^a^	5.28 ^a^	0.25
Ash	[%]	10.09 ^a^	7.55 ^b^	7.54 ^b^	0.90
Heat of combustion	[kJ kg^−1^]	16,599 ^a^	16,391 ^a^	16,385 ^a^	210
	Working state				
Transient humidity	[%]	50.08 ^a^	49.46 ^a^	49.40 ^a^	0.68
Total humidity	[%]	52.44 ^a^	52.69 ^a^	52.62 ^a^	0.31
Ash	[%]	4.93 ^a^	3.88 ^b^	3.85 ^b^	0.90
Calorific value	[kJ kg^−1^]	6369 ^b^	6389 ^a^	6390 ^a^	18,10

* The data marked with the same letters within the same parameters are not significantly different, according to Newman–Keuls multiple range test at an α level of 0.05.

## Data Availability

Data are contained within the article.
